# Magnetic Properties and Biocompatibility of Different Thickness (Pd/Fe)_n_ Coatings Deposited on Pure Ti Surface via Multi Arc Ion Plating

**DOI:** 10.3390/ma15051831

**Published:** 2022-02-28

**Authors:** Zhijun Yang, Junjie Li, Jinghua Li, Binbin Zhang, Jingxian Li, Shizhong Sheng, Peng Ding

**Affiliations:** 1 School of Mechanical and Electrical Engineering, Xinxiang University, Xinxiang 453003, China; lijingxianfff@163.com (J.L.); dingpeng@xxu.edu.cn (P.D.); 2 China Aviation Lithium Battery Technology Co., Ltd., Luoyang 471000, China; lijunjiety@163.com (J.L.); bzhfgrz@126.com (S.S.); 3 School of Medical Technology and Engineering, Henan University of Science and Technology, Luoyang 471023, China; 4 School of Mechanical and Electrical Engineering, Wenzhou University, Wenzhou 325000, China; zhangbinbin@wzu.edu.cn

**Keywords:** (Fe/Pd)_n_ coatings, pure Ti, magnetic, blood compatibility, histocompatibility

## Abstract

The different thickness (Fe/Pd)_n_ coatings were prepared by vacuum ion plating technology on a pure Ti substrate. The (Fe/Pd)_n_ coatings were magnetized using an MC-4000 high-pressure magnetizing machine. Then, the effect of the (Fe/Pd)_n_ coating thickness on the magnetic properties was studied. The surface and section morphology, composition, phase structure, magnetic properties, and biocompatibility of the (Fe/Pd)_n_ coatings were studied by scanning electron microscopy, X-ray diffraction, energy-dispersive X-ray spectroscopy, and CCTC-1 digital flux field measurement. The results showed that the (Fe/Pd)_n_ coatings were granular, smooth, and compact, without cracks. In addition the (Fe/Pd)_n_ coatings formed an L_10_ phase with a magnetic face-centered tetragonal-ordered structure after heat treatment. With the increase in the thickness of (FePd)_n_ coatings, the content of L_10_ FePd phase increased and the remanence increased. The remanence values of the Fe/Pd, (Fe/Pd)_5_, (Fe/Pd)_10_, and (Fe/Pd)_15_ magnetic coatings were 0.83 Gs, 5.52 Gs, 7.14 Gs, and 7.94 Gs, respectively. Additionally, the (Fe/Pd)_n_ magnetic coatings showed good blood compatibility and histocompatibility.

## 1. Introduction

Ti and Ti alloys have a high specific strength, low elastic modulus, good corrosion resistance, and excellent biocompatibility, and they have been widely used in aerospace, chemical, biomedical fields, etc. [1,2,3,4]. Pure Ti and Ti alloys are metal materials with good biocompatibility; they have been widely used in medicine [5,6,7,8]. At present, pure Ti and Ti alloy are some of the main materials of vascular stents [9,10]. After the introduction of metal stents into the clinic, the development of angioplasty has been promoted. However, because of the exudation of ions on the surface of metal vascular stents, the intimal hyperplasia of the inner wall of blood vessels promotes the formation of thrombosis, and postoperative restenosis still exists, which seriously affects the development of interventional heart disease [11,12].

In recent years, the biological and therapeutic effects of magnetic fields have attracted considerable attention worldwide [13,14]. Shankayi et al. found that a pulsed magnetic field not only improves local blood circulation but also has anti-inflammatory, analgesic, immunosuppressive, and drug synergistic effects [15]. Matsumoto et al. applied a constant magnetic field on canine carotid arteries that had been stripped of endothelium and found that the constant magnetic field could significantly improve the proliferation and repair of the visceral epithelial cell (VEC) [16]. Yen-patton et al. found that the weak pulsed magnetic field could promote the proliferation of VEC and accelerate the vascularization of endothelial cells in three-dimensional substrate in-vitro [17]. Many studies [18,19] have shown that a constant magnetic field can produce significant biological effects on vascular smooth muscle cells (VSMC) and endothelial cells under the action of injury factors. Constant magnetic fields with appropriate intensity can inhibit the proliferation of VSMC and promote the proliferation of VEC. The appropriate magnetic field strength of the Ti alloy stent may prevent and treat restenosis after coronary artery intervention.

FePd alloy has significant magnetic strength [20,21,22,23], providing a constant magnetic field for Ti alloy stent, which may effectively prevent vascular restenosis. Many studies on the structure and magnetic properties of FePd alloys have been conducted [24,25]. Oshima et al. [26] reported on the atomic ordering and magnetism in L_10_ ordered FePd alloys. Muto et al. [27] revealed that the temperature dependence of the tweed structure and second order elastic constants during the FCC–FCT transformation in FePd alloys. However, the preparation and magnetic properties of the Fe/Pd coatings on a pure Ti substrate are less studied. The application of Fe/Pd magnetic coatings to Ti alloy stent has the following basic problems. Whether the magnetic Fe/Pd coatings with or without cracks can be prepared on pure Ti substrate and whether the magnetic strength can be adjusted by controlling the thickness of the (Fe/Pd)_n_ coatings.

In addition, biosafety evaluation of any new biomaterial is essential before it is applied to humans [28,29,30]. The material is required to have good blood compatibility after contact with blood, so as to reduce the adverse reactions and toxic side effects caused by the material on patients; it is not toxic to cells [31,32]. Blood compatibility and in-vitro cytotoxicity assay must be considered for magnetic (Fe/Pd)_n_ coatings. 

Therefore, the (Fe/Pd)_n_ coatings were prepared on the surface of pure Ti by vacuum arc ion plating in this study. The chemical composition, microstructure, and section morphology of the (Fe/Pd)_n_ coatings prepared on the pure Ti surface were studied to analyze the quality of the (Fe/Pd)_n_/Ti coatings. Moreover, the effect of coatings thickness for the (Fe/Pd)_n_ on remanence performance was studied. Finally, the biocompatibility of the (Fe/Pd)_n_ coatings was studied by hemolysis rate test, dynamic coagulation time test, and toxicity test.

## 2. Experimental Materials and Methods

### 2.1. Preparation of the (Pd/Fe)_n_ Coatings

**Figure 1:**
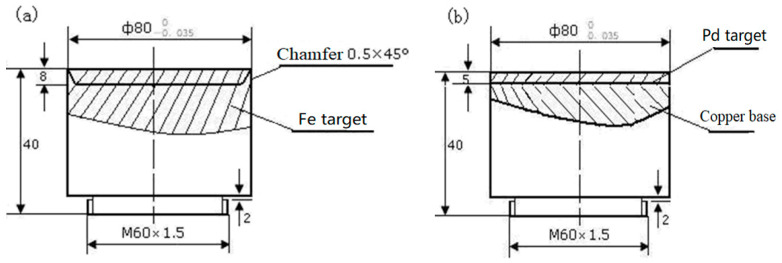
The sketch map of the Fe and Pd targets; (**a**) the Fe target and (**b**) the Pd target.

The different thickness (Pd/Fe)_n_ coatings were prepared on φ12 × 3 mm pure Ti substrate by vacuum arc ion plating. In this experiment, pure Fe target and pure Pd target with purity higher than 99.99 at.% were used to prepare the (Pd/Fe)_n_ coatings, as shown in Figure 1. The pure Ti substrate was cleaned using a metal cleaning solution; thereafter, it was ultrasonically cleaned using ethanol and placed on the substrate rack after drying. The sample was prevacuumized to 5 × 10^−3^ Pa and heated to 200 °C; afterward, the heating was stopped. Thereafter, the sample was filled with high purity argon gas (99.99%); the sample was glow-cleaned for 5–10 min. The deposition pressure was maintained at 3 × 10^−1^ Pa, the negative bias of the workpiece was 200 V (DC); the arc current was 80 A.

In order to obtain the deposition rates of Fe and Pd element, the single Fe and Pd films were deposited by vacuum arc ion plating, respectively; the film thickness was measured by weighing and scanning electron microscopy. The thickness of the deposited film was calculated by the formula:

(1) h=Δm/Sρ

where h is the deposition thickness of monolayer film, Δm is the mass of deposited metal, S is the area of deposited film, ρ is the density of deposited metal. 

The deposition rates of Fe and Pd targets were calculated by the deposition film thickness per unit of time, respectively. The calculation formula is as follows:

(2) υ=h/Δt

where υ is the deposition rate and Δt is the deposition time of single metals. According to Formulas (1) and (2), the deposition rates of Fe and Pd films are about 105 and 54 nm/min, respectively. The calculated results are in good agreement with those measured by scanning electron microscope (SEM).

The (Fe/Pd)_n_ coatings were prepared by alternating the deposition of the Fe target and Pd target by vacuum arc ion plating. The n represents the number of alternating depositions of Fe and Pd targets in the process of vacuum arc ion plating; the n is 1, 5, 10, and 15, respectively. 

For the FePd alloy formed by deposition of Fe and Pd elements, the ratio of Fe atoms to Pd atoms was about 1:1. To obtain the (Fe/Pd)_n_ coatings with ~1:1 Fe atoms to Pd atoms ratio, it is necessary to control the thickness of Fe and Pd film layers. According to the different molar quantities, densities of Fe and Pd elements, and the same surface area of the films layer, the thickness ratio of the Fe film layer to the Pd film layer is approximately 0.8:1 when Fe and Pd atoms are approximately 1 to 1, as calculated by ρV = nM and V = Hs formulas. 

The coatings thickness was controlled by the deposition time, and the deposition time of the Fe and Pd was calculated using the deposition rate, as shown in Table 1. Finally, the deposited (Fe/Pd)_n_ coatings were heated to 500 °C at a heating rate of 50 °C/min, held for 60 min, and then cooled with the furnace.

### 2.2. Characterizations and Magnetic Testing

#### 2.2.1. SEM and EDS Analysis

The surface and section morphology of the (Pd/Fe)_n_ coatings were observed with JSM-6460 scanning electron microscope (SEM); the qualitative or quantitative EDS analysis (point and plane analysis) was performed with OXFORD INCA Crystal energy dispersive spectrometer to determine the composition, content or distribution of the coatings. 

#### 2.2.2. XRD Analysis

A RIGAKU D/MAX-2400 X-ray diffractometer was used to qualitatively analyze the surface phase of some coated samples. The voltage was 40 kV, the current was 40 mA, the target was Cu, the scanning rate was 10.00 (deg/min).

#### 2.2.3. Magnetic Properties

The (Pd/Fe)_n_ coatings were magnetized using an MC-4000 high-pressure magnetizing machine. The magnetic properties of the (Pd/Fe)_n_ coatings were measured using a CCTC-1 digital flux magnetic field measuring instrument at room temperature.

### 2.3. Biocompatibility Evaluation

#### 2.3.1. Hemolysis Test

A hemolysis ratio (HR) test was performed to evaluate the release of hemoglobin under static conditions. Briefly, blood (10 mL) obtained from healthy volunteers was mixed with potassium oxalate (4.2 mL) and NaCl (5.0 mL, 0.9 wt%). Afterward, the prepared coatings were added to a NaCl solution (10.0 mL, 0.9 wt%) and incubated at 37 °C for 30 min. Next, diluted blood (0.2 mL) was added into the prepared samples (n = 5) and incubated at 37 °C for another 30 min. The liquid was centrifuged at 2500 rpm for 5 min. The optical density (OD) of the supernatant was detected using an ultraviolet–visible (UV–vis) spectrometer (Beckman DU780, USA) at a wavelength of 544 nm. The HR was evaluated according to the following relationship:

(3) $$\mathrm{HR}\left(\%\right)=({\mathrm{OD}}_{t}-{\mathrm{OD}}_{\mathrm{nc}})/({\mathrm{OD}}_{\mathrm{pc}}-{\mathrm{OD}}_{\mathrm{nc}})\times 100\%$$

where D_t_, D_nc_, and D_pc_ are the OD values of the samples.

#### 2.3.2. Coagulation Time Assay

The in-vitro anticoagulation evaluations of different coatings were carried out using an automatic coagulation analyzer (MC-2000, Beijing Mei Innovation Industry Technology Co., Ltd., Shanghai, China).

#### 2.3.3. Platelet Adhesion

To investigate the hemocompatibilities of MNPs, MNPs@CS, and MNPs@CS@Hep, platelet adhesion was measured to indicate the anticoagulation properties of the prepared magnetic adsorbents. Typically, 100 mg of series of magnetic nanoparticles were added into a 1.0 mL Platelet-rich plasma (PRP). The PRP was prepared by centrifuging (1000 rpm, 10 min) blood (10 mL, containing 3.8 wt% citrate acid solution, blood: citrate acid = 9:1). Thereafter, negative control, positive control, (Pd/Fe)/Ti, (Pd/Fe)_5_/Ti, (Pd/Fe)_10_/Ti, and (Pd/Fe)_15_/Ti were added into the PRP and incubated at 37 °C for 30 min. Next, all samples were fixed using glutaraldehyde (0.2 wt%) at 4 °C for 1 h. Subsequently, the samples were washed with phosphate-buffered saline (PBS) and dehydrated with a series of ethanol solutions (30%, 50%, 70%, 90%, and 100%). The platelet adhesion was observed using an optical microscope.

#### 2.3.4. In-Vitro Cytotoxicity Assay

MTT assay was used to determine the cytotoxicity of the pure Ti and (Pd/Fe)_n_/Ti, which were divided into experimental (Pd/Fe)_n_/Ti (n = 1, 5, 10, and 15), positive control (pure lead), and negative control (pure Ti) groups. L929 cells at a logarithmic growth stage were prepared into 1 × 10^5^ cell suspensions/mL using a medium and injected into 96-well plastic petri dishes at 300 mL each with 3 culture plates in total. At least 8 wells in each group were placed in a cell culture box at 37 °C and 5% CO_2_ for 24 h; afterward, the original medium was discarded and washed twice with PBS solution. Further, 100 mL of the (Pd/Fe)_n_/Ti (n = 1, 5, 10, and 15) extract was added to the experimental group, 100 mL of the pure lead extract was added to the positive control group, and 100 mL of the pure Ti extract was added to the negative control group. Afterward, they were placed in the above culture environment for continued cultivation for 2, 4, and 6 d.

The extract and culture medium in the petri dish were discarded; 20 μL of MTT solution was added to each well and incubated for 6 h. The MMT was sucked out, 150 μL of dimethyl sulfoxide (DMSO) was added; the crystals were fully dissolved by shock for 10 min. The absorption value of each well was measured by enzyme-linked immunosorbent assay at 540 nm; cell viability was calculated as follows:

(4) $$\mathrm{Viability}=({\mathrm{OD}}_{t}/{\mathrm{OD}}_{\mathrm{nc}})\times 100\%$$

## 3. Results and Discussion

### 3.1. Characteristics of the (Pd/Fe)_5_ Coatings

**Figure 2:**
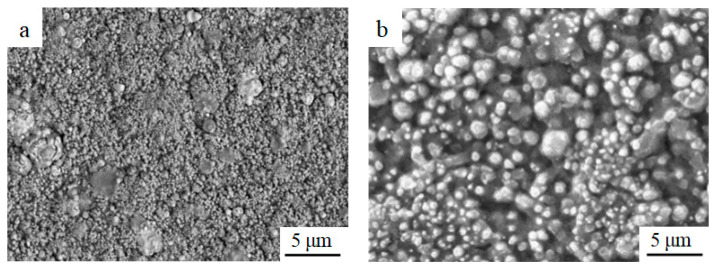
Surface morphology of the (Pd/Fe)_5_ coatings; (**a**) the sedimentary and (**b**) heat treatment state.

Figure 2a shows the surface topography of the deposition state of the (Pd/Fe)_5_ coating on the pure Ti substrate; the coating surface exhibited granular characteristics. The surface of the coating was smooth and compact without cracks. Spherical droplets of different sizes were dispersed on the surface of the coating; there were small pits formed by plasma action, which is a common feature in arc ion plating [33]. Figure 2b shows the surface morphology of the (Pd/Fe)_5_ coating after heat treatment. Small droplets disappeared, pits decreased, particles grew, and a large number of droplets existed on the surface. The morphology of these droplets changed from a small spherical shape to an irregular shape. This was due to the different atomic migration rates of the different elements at the high temperature, resulting in the formation of numerous vacancies and reverse migrations along them.

**Figure 3:**
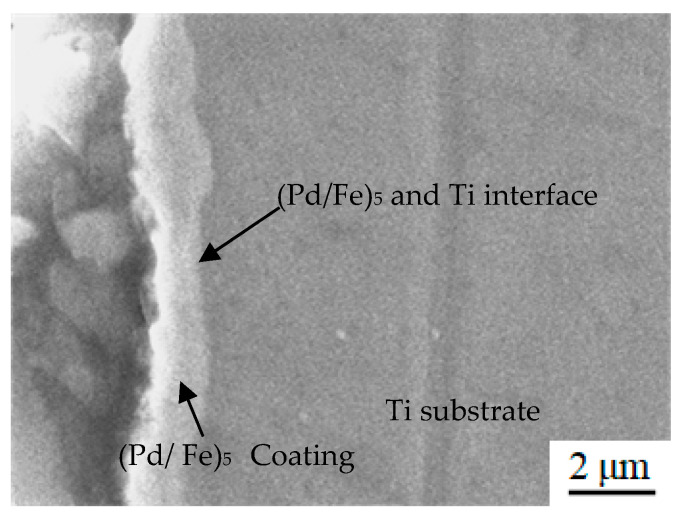
Section morphology of the (Pd/Fe)_5_/Ti coating.

Figure 3 shows the cross-sectional morphology of the (Pd/Fe)_5_/Ti coating after heat treatment. The coating thickness was approximately 1.6 μm. There were no cracks and porosity between the (Pd/Fe)_5_ coating and the Ti substrate; the bond was good. In addition, there was no obvious interface between Fe film and Pd film. However, the interface between the (Fe/Pd)_5_ coating and the Ti substrate are very clear. 

**Figure 4:**
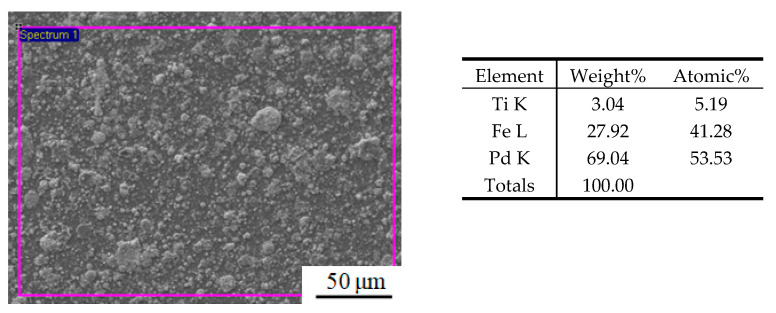
EDS energy spectrum of the (Pd/Fe)_5_/Ti surface.

Figure 4 shows the EDS energy spectrum of the (Pd/Fe)_5_/Ti surface after heat treatment. The weight percentage content of Pd was 69.04%, and that of Fe was 27.92%. There is a small amount of Ti element on the surface of the sample. The depth of penetration of the electron beam in the EDS method is about 2 μm. The thickness of the (Pd/Fe)_5_ coating is approximately 1.6 μm. Thus, Ti element is from the pure Ti substrate.

**Figure 5:**
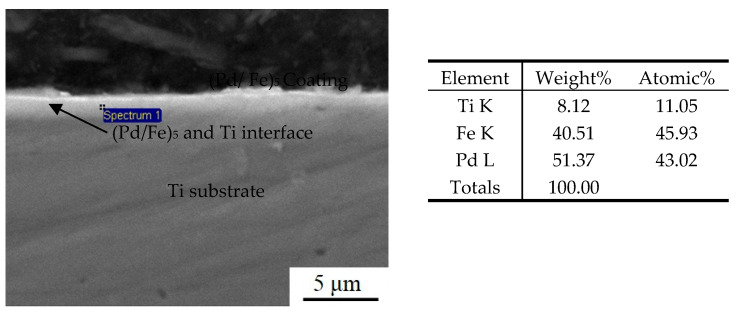
EDS energy spectrum of the section for the (Pd/Fe)_5_/Ti coating.

Figure 5 shows the EDS energy spectrum of the section for the (Pd/Fe)_5_/Ti coating after heat treatment. The weight percentage content of Ti, Fe, and Pd element were 8.12%, 40.51%, and 51.37%, respectively. The same EDS spectrum is found in different regions of the coating, indicating that the coating is homogeneous.

**Figure 6:**
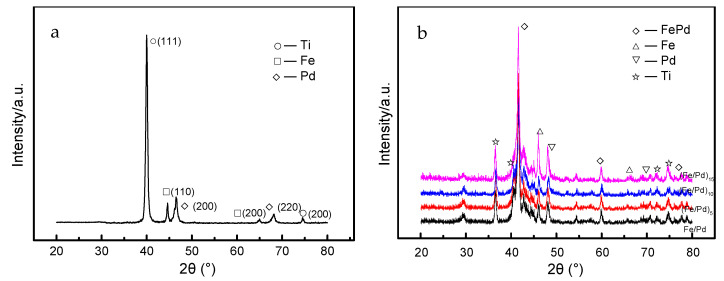
XRD patterns of the (Fe/Pd)_n_ coatings at deposition and heat treatment state; (**a**) (Fe/Pd)_5_/Ti coatings at deposition state, (**b**) Fe/Pd, (Fe/Pd)_5_, (Fe/Pd)_10_, and (Fe/Pd)_15_ coatings at heat treatment state.

Figure 6a,b show the XRD patterns of the (Pd/Fe)_n_ coatings at deposition and heat treatment state. As shown in Figure 6a, without heat treatment, the deposition state of the (Pd/Fe)_5_ coatings was mainly composed of the Pd phase, Fe phase, and Ti phase. The diffraction peaks of the Ti phase from the substrate were (111) and (200). The diffraction peaks of the Fe phase were (110) and (200), the diffraction peak of the Pd phase was (200) and (220). As shown in Figure 6b, the XRD patterns of different (Fe/Pd)_n_ coating were similar. The (Pd/Fe)_n_ coatings were mainly composed of the FePd phase, Ti phase, Pd phase, and Fe phase after heat treatment. The (110), (200), and (112) diffraction peaks of the FCT structure of the FePd alloy were observed. This indicated that the FePd phase of the L_10_ ordered phase with hard magnetic FCT structure was formed at a high temperature. That is, the internal structure of the Fe/Pd coating changed from the Fe phase and Pd phase to the ordered FCT phase after heat treatment and from superparamagnetic to ferromagnetic. 

### 3.2. Surface and Section Morphology of the (Pd/Fe)_n_ Coatings

**Figure 7:**
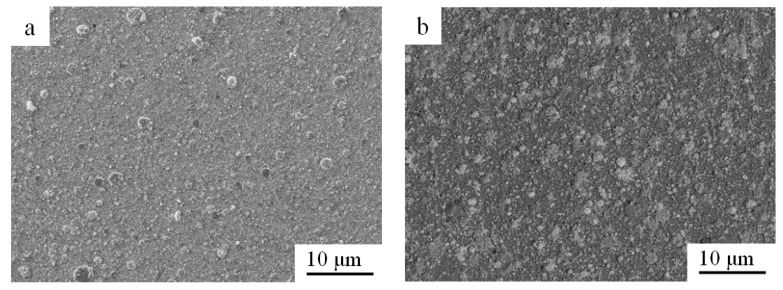

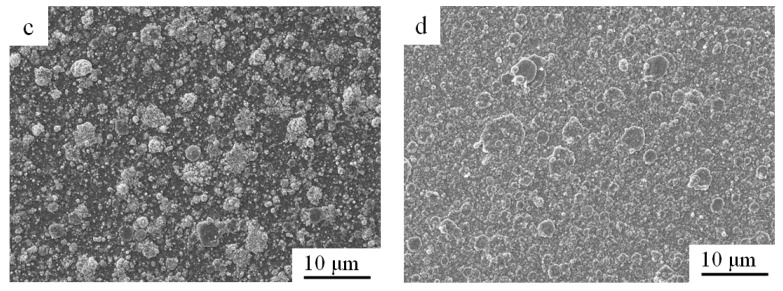
Surface morphology of different (Fe/Pd)_n_/Ti coatings after heat treatment; (**a**) (Fe/Pd)/Ti, (**b**) (Fe/Pd)_5_/Ti, (**c**) (Fe/Pd)_10_/Ti, and (**d**) (Fe/Pd)_15_/Ti.

Figure 7 shows the SEM surface morphology of the (Fe/Pd)/Ti, (Fe/Pd)_5_/Ti, (Fe/Pd)_10_/Ti, and (Fe/Pd)_15_/Ti after heat treatment. As shown in Figure 7a, there are few “large droplets” on the surface of the Fe/Pd monolayer, with a particle diameter of approximately 5 μm and the largest particle diameter of 10 μm; further, the distribution is sparse. As shown in Figure 7b–d, as the number of Fe/Pd film layers increases, the size of the “droplets” increases, and several droplet particles fuse to form larger “droplets.” The formation of such large droplet particles was due to the numerous droplets vaporized by the Pd and Fe ions in the arc ion plating process. Many droplets met on the surface of the substrate and fused into a large droplet particle because of the high temperature. With the increase in the number of Fe/Pd film layers, the droplet size and the surface roughness increased. The largest droplets on the (Fe/Pd)_15_/Ti surface were 10–25 μm in size.

**Figure 8:**
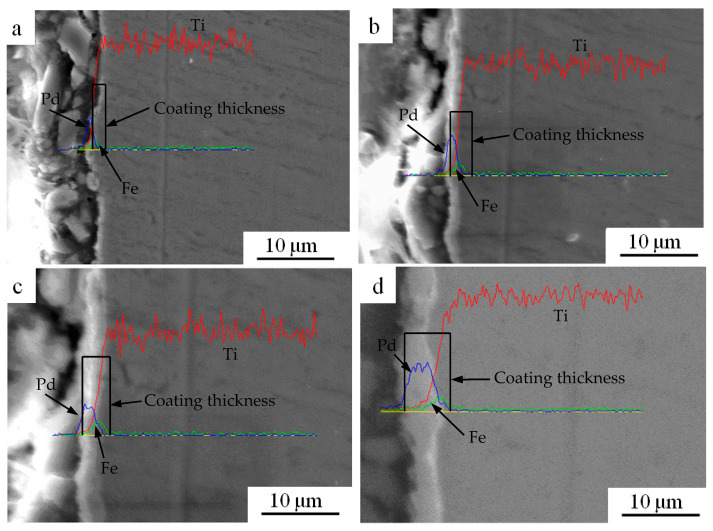
SEM photographs of cross-sectional microstructure for the (Fe/Pd)_n_/Ti coating after heat treatment; (**a**) (Fe/Pd)/Ti, (**b**) (Fe/Pd)_5_/Ti, (**c**) (Fe/Pd)_10_/Ti, and (**d**) (Fe/Pd)_15_/Ti.

Figure 8 shows the morphology of cross-sectional microstructure for the (Fe/Pd)_n_/Ti coatings after heat treatment. As shown in Figure 8a, the monolayer Fe/Pd formed a continuous coating, and the thickness of the Fe/Pd coating was approximately 0.4 μm. As shown in Figure 8b–d, as the number of film layers increased, the thickness of the Fe/Pd coating increased. The thicknesses of the Fe/Pd coating of the (Fe/Pd)_5_/Ti, (Fe/Pd)_10_/Ti, and (Fe/Pd)_15_/Ti coating were approximately 1.8 μm, 3.5 μm, and 5.5 μm, respectively, which was consistent with the theoretical calculation of the coating thickness. Uniform (Fe/Pd)_n_ coatings can be deposited on the surface of a pure Ti substrate by vacuum arc ion plating technology. Fe and Pd ions in the deposition state have enough energy; thus, the Fe and Pd layers were well bonded. After heat treatment, FePd alloy was formed between Fe film and Pd film without obvious interface. Additionally, the thickness of Fe/Pd coating thickness increased with the increase in the number of the Fe/Pd film layers; the content of the FePd alloy also increased, which is conducive to increasing the remanence value of the (Fe/Pd)_n_ coatings. Thus, (Fe/Pd)_n_ coatings with different remanence can be afforded by adjusting the number of alternating deposition of the Fe and Pd element.

### 3.3. Magnetic Analysis of the (Pd/Fe)_n_ Coatings

Table 2 shows the remanence value of the (Fe/Pd)_n_/Ti samples on the surface, 1 mm and 2 mm away from the surface after heat treatment. The pure Ti substrate was not magnetic. Without heat treatment, the surface remanence values of Fe/Pd, (Fe/Pd)_5_, (Fe/Pd)_10_, and (Fe/Pd)_15_ coatings were 0.12 Gs, 0.18 Gs, 0.23 Gs, and 0.24 Gs, respectively. The remanence value is very small. After heat treatment, the magnetic properties of (Fe/Pd)_n_ coatings were greatly increased. In addition, the (Fe/Pd)/Ti sample was magnetically weak, and the surface remanence value was only 0.83 Gs. When the Fe and Pd film layers reached 5, the surface remanence value of the (Fe/Pd)_5_/Ti sample significantly increased; the remanence value was approximately 5.52 Gs, which was approximately 6 times that of the (Fe/Pd)/Ti samples. The remanence value of the (Fe/Pd)_10_/Ti sample was approximately 7.14 Gs, which was 1.62 Gs higher than that of the (Fe/Pd)_5_/Ti sample. The remanence value of the (Fe/Pd)_15_/Ti was approximately 7.91 Gs, and the magnetism increased by 0.8 Gs compared with the (Fe/Pd)_10_/Ti. With the increase in the number of film layers, the remanence curvilinearly increased; however, the increase amplitude decreased.

Compared with the surface remanence value, the remanence value of the (Fe/Pd)_n_/Ti coating at 1 mm and 2 mm from the surface had the same variation law; however, it gradually decreased with the increase in the distance from the surface of the (Fe/Pd)_n/_Ti coating. The remanence value of the monolayers at 1 mm and 2 mm away from the surface of the (Fe/Pd)/Ti monolayers was only 0.63 Gs and 0.31 Gs, respectively. When the Fe/Pd film reached 15 layers on the pure Ti substrate, the remanence value of the (Fe/Pd)_15_/Ti sample at 1 mm and 2 mm away from the surface of the coating was approximately 5.22 Gs and 3.91 Gs, respectively.

In the deposition state, the (Fe/Pd)_n_ coating with only the Fe and Pd phase is not magnetic. After heat treatment, the internal particle structure of the (Fe/Pd)_n_ coating changes from the Fe and Pd phase to the chemically ordered FCT phase with tetragonal lattice; thus, the internal particle structure changes from superparamagnetic to ferromagnetic. The (Fe/Pd)_n_ coatings is magnetic, and the remanence increases with the increase in the FCT phase content. In the tetragonal lattice, Fe and Pd atoms occupy the (001) plane of the FCT lattice, forming an ordered structure with ferromagnetism, which is also known as the L_10_ phase in metallurgy. However, L_10_ phase has very high magnetocrystalline anisotropy, which can provide the coercivity required to form permanent magnets.

The mechanism of the magnetic action is as follows. Due to the strong “exchange” between the electron spin magnetic moments of the adjacent magnetic atoms of the Fe and Pd particles, the adjacent electron magnetic moments are arranged in a specific way. If the exchange is positive, these atomic magnetic moments are aligned parallel to each other, resulting in numerous magnetic moments. It is the ordered arrangement of the magnetic moments of the atoms in the (Fe/Pd)_n_ coating that produces ferromagnetism. Magneto-crystal exchange coupling is when two adjacent grains undergo direct-contact interface-exchange coupling cooperation with different orientations of magnetic moments. To prevent its magnetic moment along the respective preferred direction orientation, the magnetic moment of an orientation of the interface from one easy magnetization direction of the grain continuously changes for another easy magnetization direction of the grain. The grain magnetic moment of chaos orientation favors parallel arrangement. Resultantly, the component of the magnetic moment along the direction of external magnetic fields increases, resulting in a remanent magnetic enhancement effect.

### 3.4. Biocompatibility of the (Pd/Fe)_n_ Coatings

#### 3.4.1. Blood Compatibility

The HRs test is an acute cytotoxicity test that was performed in-vitro using cells cultured from fresh rabbit blood [34,35]. It is generally believed that if the HR of a material is less than 5%, the material meets the hemolysis requirements of medical materials. If the HR is >5%, the material has hemolytic effects [36,37]. To investigate the blood compatibilities of the as-prepared coating, we measured their HRs, coagulation times, and platelet adhesions (Table 3). 

The HRs of the positive control, negative control, pure Ti group, (Pd/Fe)/Ti, (Pd/Fe)_5_/Ti, (Pd/Fe)_10_/Ti, and (Pd/Fe)_15_/Ti were 3.24 ± 0.44, 3.58 ± 0.87, 3.78 ± 0.23, 3.25 ± 0.41, 3.36 ± 0.78, 3.14 ± 0.23, and 2.64 ± 0.45, respectively; they were less than the limit (5%). Therefore, neither of the Ti and (Fe/Pd)_n_ materials will cause acute hemolysis. Further, platelets were not significantly activated, indicating that the well-prepared magnetic nanoparticles had good blood compatibility. This implies that those magnetic adsorbents can be directly applied in clinics. The anticoagulation of the different samples was evaluated in-vitro using prothrombin time (PT), activated partial thromboplastin time (APTT), and thrombin time (TT). The (Pd/Fe)_15_/Ti nanoparticles displayed highly increased PT, APTT, and TT (10.7 ± 1.5, 32.5 ± 4.3, and 13.2 ± 0.8, respectively) when compared with the positive control (11.5 ± 1.6, 33.3 ± 4.7, and 13.5 ± 0.7) and the negative control (9.1 ± 2.1, 28.2 ± 3.5, 9.3 ± 2.5, respectively).

#### 3.4.2. Platelet Adhesion

**Figure 9:**
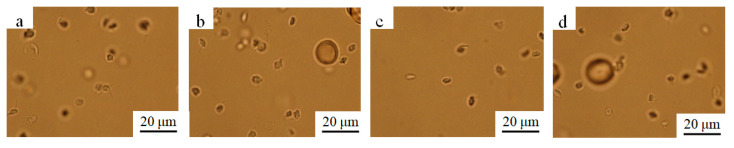

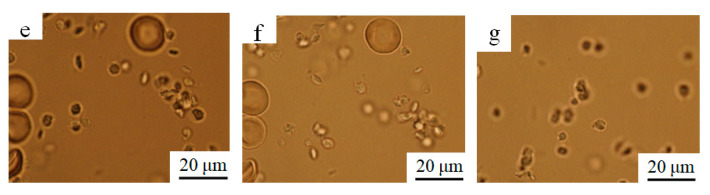
Optical images of the platelets adhesion; (**a**) negative control, (**b**) positive control, (**c**) pure Ti group, (**d**) (Pd/Fe)/Ti, (**e**) (Pd/Fe)_5_/Ti, (**f**) (Pd/Fe)_10_/Ti, and (**g**) (Pd/Fe)_15_/Ti; Temperature: 37 °C; Time: 30 min; pH: 7.4.

Platelet adhesion assay suggested that the (Pd/Fe)_15_/Ti coating had superior property to that of the control group. Figure 9 shows the optical micrographs of the platelet adhesion for every group. After adding the different coating, particularly for (Pd/Fe)_15_/Ti (Figure 9g), platelets were not significantly activated, which suggests that the well-prepared magnetic nanoparticles had good blood compatibility.

#### 3.4.3. Cytotoxicity Test

**Figure 10:**
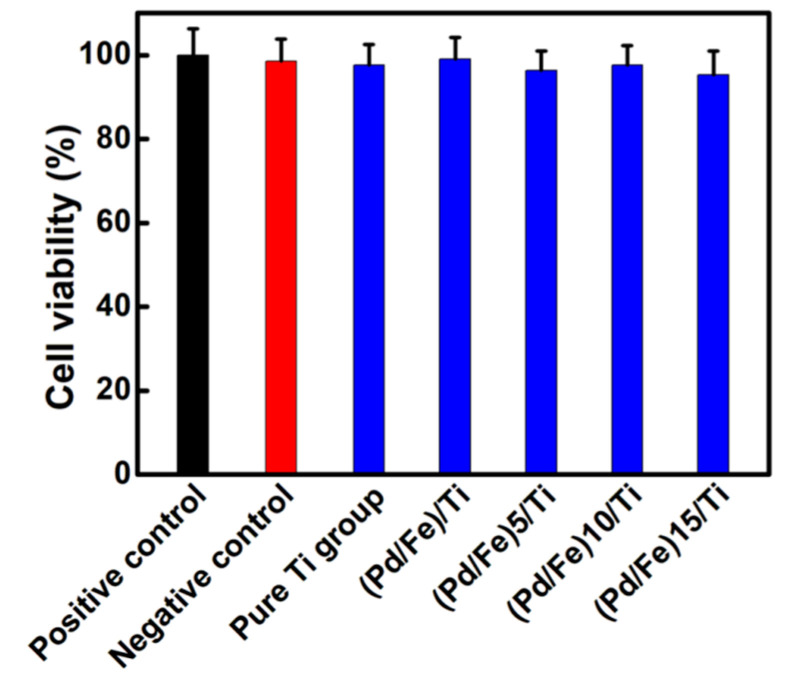
Cell toxicity evaluation of the coating.

Figure 10 shows the cell relative cytotoxicity of the positive control group, negative control group, and (Pd/Fe)_n_/Ti (n = 1, 5, 10, and 15) group by a typical MTT experiment. Significant cell death was not observed for the cells treated with the (Pd/Fe)_n_ coating (survival rate: above 90%), and showed an increasing trend with the increase in time. The (Fe/Pd)_n_ magnetic coating showed no toxicity to the cells. This indicates that the (Fe/Pd)_n_ magnetic coating had good biocompatibility with the L929 cells and no cytotoxicity. All results showed that the coating was considerably biocompatible for further application.

**Figure 11:**
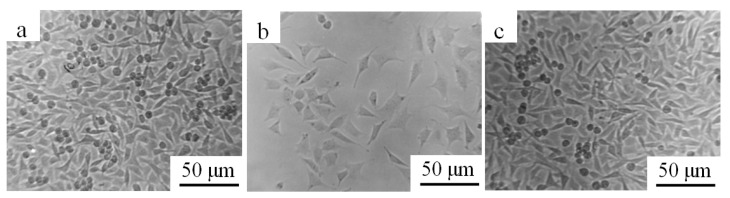
Cell morphology photos of each group for 4 d; (**a**) negative control group, (**b**) positive control group, and (**c**) (Pd/Fe)_15_ group.

Figure 11 shows the cell morphology photos of the negative control group, positive control group, and (Pd/Fe)_15_ group for 4 d. The number of cells in the positive control group (pure lead) was significantly lower than that in the negative control group (pure Ti); the cells became round and small. The number of cells on the surface of the (Pd/Fe)_15_ sample was significantly higher than that on the surface of the positive control group, which was similar to that on the surface of the negative control group, indicating that the Pd/Fe coating did not increase the cytotoxicity of the pure Ti substrate.

## 4. Conclusions

The different thickness (Pd/Fe)_n_ coatings were prepared by vacuum ion plating technology on a pure Ti substrate and evaluated as a potential vascular scaffold material for biomedical applications. The results can be summarized as follows:

1. The (Fe/Pd)_n_ coatings can be continuously distributed on the surface of the pure Ti substrate by vacuum ion plating technology. The surface of the coating is smooth and compact, without cracks, and has good bonding with the pure Ti substrate.
2. The (Fe/Pd)_n_ coatings formed the L_10_ FePd phase with magnetic FCT ordered structure after heat treatment; the (Fe/Pd)_n_ coatings obtained magnetism.
3. The Fe/Pd coating is magnetically weak, and the remanence of the coating surface is only 0.83 Gs. With the increase in the thickness of the (FePd)_n_ coating layers, the content of the FePd phase in the FCT structure increases and the remanence increases. The remanence values of the (Fe/Pd)_5_, (Fe/Pd)_10_, and (Fe/Pd)_15_ magnetic coatings are approximately 5.52 Gs, 7.14 Gs, and 7.94 Gs, respectively.
4. The HRs of the (Pd/Fe)_n_ magnetic coatings were lower than the limit (5%); platelets were not significantly activated, indicating that the prepared magnetic nanoparticles have good blood compatibility. No significant cell death was observed for the cells treated with the (Pd/Fe)_n_ magnetic coatings, indicating that the (Fe/Pd)_n_ magnetic coatings had no cytotoxicity.

## Figures and Tables

**Table 1:** Deposition time of the (Fe/Pd)_n_ coatings.

Sample No.	Fe Time (s)	Pd Time (s)	Atomic Ratio (Fe:Pd )	Thickness Ratio (Fe:Pd )	n (Times)	Total Thickness (μm)
1	72 s	185 s	1:1	0.8:1	1	0.3
2	72 s	185 s	1:1	0.8:1	5	1.5
3	72 s	185 s	1:1	0.8:1	10	3
4	72 s	185 s	1:1	0.8:1	15	4.5

**Table 2:** The magnetic properties of the (Fe/Pd)_n_/Ti samples.

Distance from Surface	Remanence/(Gs)				
	Pure	Fe/Pd	(Fe/Pd)_5_	(Fe/Pd)_10_	(Fe/Pd)_15_
Surface	0	0.83	5.52	7.14	7.94
1 mm	0	0.63	3.95	4.85	5.22
2 mm	0	0.31	2.23	3.53	3.91

**Table 3:** The HR and anticoagulation evaluations of the positive control group, negative control group, pure Ti group, and (Pd/Fe)n/Ti (n = 1, 5, 10, and 15) group.

Group	HR(%)	PT(s)	APTT(s)	TT(s)
Positive control	3.24 ± 0.44	11.5 ± 1.6	33.3 ± 4.7	13.5 ± 0.7
Negative control	3.58 ± 0.87	9.1 ± 2.1	28.2 ± 3.5	9.3 ± 2.5
Pure Ti group	3.78 ± 0.23	9.7 ± 1.9	28.1 ± 2.7	9.4 ± 2.6
(Pd/Fe)/Ti	3.25 ± 0.41	10.9 ± 1.8	32.7 ± 4.6	13.2 ± 1.4
(Pd/Fe)_5_/Ti	3.36 ± 0.78	10.4 ± 2.2	28.5 ± 3.6	9.4 ± 2.8
(Pd/Fe)_10_/Ti	3.14 ± 0.23	10.4 ± 2.5	29.9 ± 2.7	9.6 ± 2.4
(Pd/Fe)_15_/Ti	2.64 ± 0.45	10.7 ± 1.5	32.5 ± 4.3	13.2 ± 0.8
